# The construction of a predictive model for postoperative bladder paralysis in patients with cervical cancer based on logistic regression and nomogram

**DOI:** 10.3389/fonc.2026.1877348

**Published:** 2026-09-18

**Authors:** Lishan Huang, Weihong Zeng, Ru Pan, Zhuying Yu, Haihong Lin, Haikun Yang

**Affiliations:** Department of Gynecology, Meizhou People’s Hospital, Meizhou, Guangdong, China

**Keywords:** bladder paralysis, cervical cancer, influencing factors, nomogram, radical hysterectomy

## Abstract

**Objective:**

To construct a predictive model for postoperative bladder paralysis in patients with cervical cancer (CC) based on Logistic regression and nomogram.

**Methods:**

The clinical data of 220 patients with CC admitted to our hospital from December 2019 to September 2022 were retrospectively collected as the modeling cohort. In addition, the clinical data of 130 patients with CC admitted to the same hospital during a later time period, from October 2022 to September 2025, were collected as the temporal validation cohort. According to whether the patients in the modeling and validation cohorts developed bladder paralysis after surgery, they were separated into the bladder paralysis groups and the non-bladder paralysis groups.

**Results:**

Logistic regression analysis of the variables selected by LASSO regression showed that clinical staging, number of lymph nodes removed, extent of hysterectomy, width of parametrial tissue resection, and vaginal stump length were risk factors for postoperative bladder paralysis in patients with CC (P < 0.05). The AUC in the modeling cohort was 0.899, and 0.899, and the H-L test yielded χ^2^ = 7.405 (P = 0.734). DCA showed a favorable net clinical benefit across threshold probabilities of 0.14–0.82. In the temporal validation cohort,the AUC was 0.944, and the H-L test yielded χ^2^ = 7.234 (P = 0.725). DCA showed a favorable net clinical benefit across threshold probabilities of 0.19–0.84.

**Conclusion:**

Clinical staging, number of lymph nodes removed, extent of hysterectomy, width of parametrial tissue resection, and vaginal stump length were risk factors for postoperative bladder paralysis in patients with CC. The nomogram model constructed based on Logistic regression has good consistency and discrimination.

## 1 Introduction

Cervical cancer (CC), as a common gynecological malignancy, ranks second among tumors of the female reproductive system. Its pathogenesis remains unclear, but may be associated with high-risk human papillomavirus infection, genetic factors, and others. Patients may present with vaginal bleeding in the early to middle stages, while anemia, urinary urgency, and lower limb swelling occur in the late stage, often leading to poor prognosis. In recent years, the incidence of CC has remained high and shows a trend toward younger age at onset (1, 2). For patients with early-stage CC, radical surgery is the first-line clinical treatment, aiming to completely remove the tumor lesions and thereby prolong survival. However, such surgical procedures are highly invasive, and some patients may experience postoperative bladder paralysis, manifested mainly as urinary incontinence and dysuria, which negatively affects quality of life (3–5). Therefore, this study investigates the influencing factors of postoperative bladder paralysis in CC patients and aims to improve prognosis by modifying these risk factors. A nomogram can be constructed based on risk factors identified through logistic regression analysis to evaluate the probability of adverse events, thus assisting clinicians in targeted prevention (6, 7). At present, few studies have reported the application of nomograms in predicting postoperative bladder paralysis in CC patients. Therefore, this study seeks to establish a predictive model for postoperative bladder paralysis in CC patients using logistic regression and nomogram construction.

## 2 Materials and methods

### 2.1 General information

Clinical data of 220 CC patients treated in our hospital from December 2019 to September 2022 were retrospectively collected as the modeling group. In addition, data of 130 CC patients treated at the same hospital during a subsequent time period, from October 2022 to September 2025, were collected as the temporal validation cohort. Both cohorts were further divided into a bladder paralysis group and a non-bladder paralysis group according to whether postoperative bladder paralysis occurred. Clinical staging was determined according to the 2018 International Federation of Gynecology and Obstetrics (FIGO) staging system (8). The case collection flowchart is shown in **Figure 1**. Inclusion criteria: (1) Met the diagnostic criteria for CC (8); postoperative pathological confirmation; (2) first-time surgical treatment; (3) age > 18 years; (4) complete clinical data available. Exclusion criteria: (1) history of malignancy; (2) urinary system calculi; (3) pre-existing urinary dysfunction; (4) hematologic disorders; (5) hepatic or renal dysfunction; (6) history of pelvic surgery; (7) infectious diseases. This study was approved by the hospital ethics committee.

**Figure 1:**
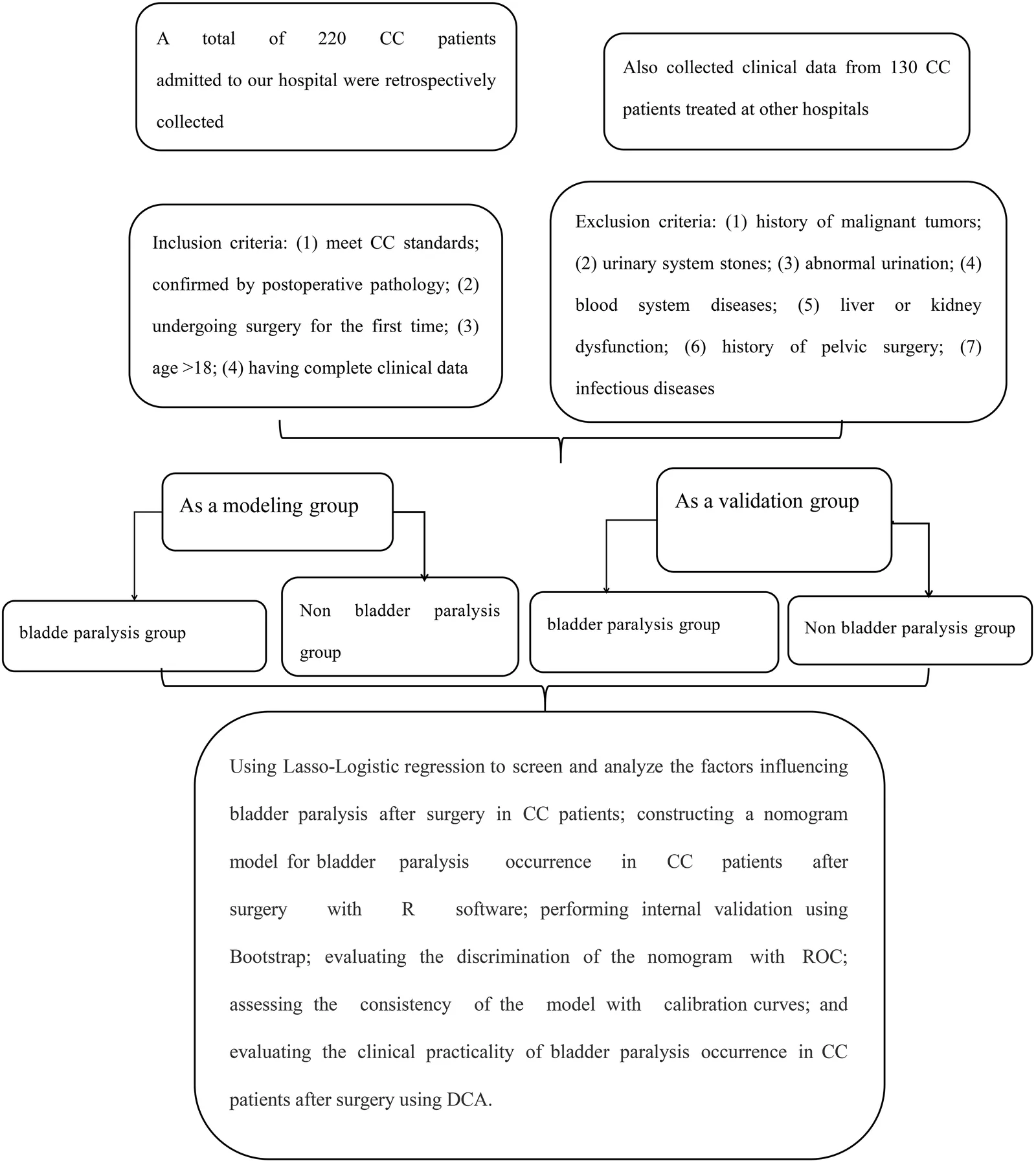
Case flow collection diagram.

### 2.2 Methods

#### 2.2.1 Treatment methods

According to relevant guidelines (9), patients with stage IA underwent cervical hysterectomy (modified radical hysterectomy, which can also be performed extrafascially, with resection of only part of the parametrial tissue, transection of the cardinal and uterosacral ligaments close to the uterus, resection of 1–2 cm of the vagina, and pelvic lymph node management). Patients with stages IB–IIB underwent radical hysterectomy with resection of ≥3 cm of parametrial tissue and ≥3 cm of the vagina, combined with pelvic lymph node dissection or sentinel lymph node assessment. If postoperative pathology indicated risk factors, adjuvant chemoradiotherapy could be administered. For patients with stage IIB disease, radical surgery was performed only after multidisciplinary gynecologic oncology assessment confirmed localized parametrial involvement without pelvic wall invasion and adequate physical fitness to tolerate surgery. All surgical procedures were performed by senior gynecologic oncology surgeons. The surgical approaches included laparoscopy and laparotomy, and pelvic autonomic nerve-sparing surgery was performed according to the tumor characteristics. During nerve-sparing procedures, the hypogastric nerves, pelvic splanchnic nerves, and pelvic plexus were identified and preserved.

#### 2.2.2 Diagnostic criteria for bladder paralysis

Patients were followed up for one month postoperatively, with the assessment performed at 1 month after surgery. Bladder paralysis was determined based on a comprehensive evaluation of subjective lower urinary tract symptoms (including incomplete bladder emptying, dysuria, urinary retention, urinary incontinence, and decreased bladder sensation), post-void residual urine volume measured by urinary ultrasonography, and the need for catheterization-assisted voiding. A post-void residual urine volume ≥100 mL and/or the need for catheterization intervention (10) was used as the objective diagnostic criterion for bladder paralysis. The outcome was assessed independently by two uniformly trained urologists, and any disagreement was resolved through joint review. In addition, patients with urinary tract infection were explicitly excluded to avoid interference from infection-related urinary symptoms in outcome assessment.

#### 2.2.3 Collection of clinical data

Clinical data were retrieved from patients’ electronic medical records, including: age, BMI, diabetes, hypertension, pathological type, clinical stage, preoperative chemoradiotherapy, number of lymph nodes removed, depth of cervical stromal invasion, intraoperative blood loss, operation time, extent of hysterectomy, width of parametrial resection (measured as the distance from the uterine margin to the tissue resection margin along the cardinal and uterosacral ligaments, with the mean of the bilateral measurements used for analysis), vaginal stump length (measured as the length of the resected vaginal wall specimen from the vaginal fornix to the vaginal resection margin), and postoperative hospital stay. For all variables with a missing-data rate <20%, multiple imputation by chained equations (MICE) was performed. Variables with a missing-data rate ≥20%, which were considered to have a high risk of data distortion, were excluded to maximize sample retention and reduce bias; however, no variables were excluded on this basis in the present study.

### 2.3 Statistical analysis

Data were analyzed using SPSS 25.0. Categorical variables were expressed as n (%) and compared with the χ² test. Continuous variables were expressed as mean $\left(\bar{x}\pm s\right)$ and compared with the t-test. Variables showing statistically significant differences in the univariable analysis were initially considered candidate predictors. LASSO regression was subsequently performed for further variable shrinkage and selection. Variables retained by LASSO were then simultaneously entered into a multivariable logistic regression model to identify independent factors associated with postoperative bladder paralysis. No forward stepwise selection procedure was used. R software was used to construct the nomogram model. Internal validation was performed using the bootstrap method. The discriminatory ability of the nomogram was evaluated using ROC curves; calibration was assessed with calibration plots; and clinical utility was assessed using decision curve analysis (DCA). A p-value < 0.05 was considered statistically significant.

## 3 Results

### 3.1 Comparison of clinical data between the bladder paralysis group and the non-bladder paralysis group in the modeling cohort

Among the 220 patients in the modeling cohort, 67 developed bladder paralysis (30.45%). Significant differences were observed between the bladder paralysis group and the non-bladder paralysis group in terms of clinical stage, postoperative chemoradiotherapy, number of lymph nodes removed, depth of cervical stromal invasion, extent of hysterectomy, width of parametrial resection, and length of the vaginal stump (P<0.05). No significant differences were observed for other baseline characteristics (P > 0.05) (**Table 1**).

**Table 1:** Comparison of clinical data between the bladder paralysis group and non-bladder paralysis group in the modeling group.

Factor	Bladder paralysis group (n=67)	Non bladder paralysis group (n=153)	*χ* ^2^	*P*
Age			0.859	0.354
≥50 years old	44 (65.67)	110 (71.90)		
<50 years old	23 (34.33)	43 (28.10)		
BMI			3.136	0.077
≥24 kg/m^2^	20 (29.85)	65 (42.48)		
<24 kg/m^2^	47 (70.15)	88 (57.52)		
Diabetes			0.043	0.835
Yes	9 (13.43)	19 (12.42)		
No	58 (86.57)	134 (87.58)		
Hypertension			2.781	0.095
Yes	11 (16.42)	41 (26.80)		
No	56 (83.58)	112 (73.20)		
Pathological type			0.021	0.886
Squamous cell carcinoma	57 (85.07)	129 (84.31)		
Adenocarcinoma	10 (14.93)	24 (15.69)		
Clinical staging			18.218	<0.001
IA~IB	30 (44.78)	114 (74.51)		
IIA~IIB	37 (55.22)	39 (25.49)		
Postoperative chemoradiotherapy			13.769	<0.001
yes	54 (80.60)	83 (54.25)		
no	13 (19.40)	70 (45.75)		
Number of lymph nodes removed (pcs)			5.461	0.019
≥25	52 (77.61)	94 (61.44)		
<25	15 (22.39)	59 (38.56)		
Depth of cervical stromal invasion			6.126	0.013
≥1/2 of the cervical stroma	53 (79.10)	95 (62.09)		
<1/2 of the cervical stroma	14 (20.90)	58 (37.91)		
Amount of bleeding during surgery (mL)			1.189	0.275
≥50	35 (52.24)	92 (60.13)		
<50	32 (47.76)	61 (39.87)		
Surgery time (min)			1.291	0.256
≥180	44 (65.67)	88 (57.52)		
<180	23 (34.33)	65 (42.48)		
Extent of hysterectomy			7.300	0.007
Radical hysterectomy	54 (80.60)	95 (62.09)		
Modified radical hysterectomy	13 (19.40)	58 (37.91)		
Width of parametrial tissue resection (cm)			31.496	<0.001
≥3	41 (61.19)	34 (22.22)		
<3	26 (38.81)	119 (77.78)		
Vaginal stump length (cm)			26.671	<0.001
≥3	37 (55.22)	31 (20.26)		
<3	30 (44.78)	122 (79.74)		
Postoperative hospital stay (d)			1.871	0.171
≥7	32 (47.76)	58 (37.91)		
<7	35 (52.24)	95 (62.09)		

### 3.2 Lasso-logistic regression analysis of risk factors for postoperative bladder paralysis in CC patients

Occurrence of postoperative bladder paralysis (yes = 1, no = 0) in the modeling cohort was set as the dependent variable, while factors showing significant differences in **Table 1** were initially considered candidate predictors for LASSO regression: clinical stage (IA–IB = 0, IIA–IIB = 1), Postoperative chemoradiotherapy (yes = 1, no = 0),number of lymph nodes removed (≥25 = 1, <25 = 0), depth of cervical stromal invasion (≥1/2 of the cervical stroma=1, <1/2 of the cervical stroma=0),extent of hysterectomy (radical hysterectomy = 1, modified radical hysterectomy = 0), width of parametrial resection (≥ 3 cm = 1, < 3 cm = 0), and vaginal stump length (≥ 3 cm = 1, < 3 cm = 0). Using R software for Lasso analysis, results showed that the model performed best when λ = 0.0610128. Ultimately, five predictive factors were retained: clinical stage, number of lymph nodes removed, extent of hysterectomy, width of parametrial resection, and vaginal stump length(**Figure 2**). Multicollinearity analysis showed that the variance inflation factors of the above variables were <5, indicating no multicollinearity. These five variables were then simultaneously entered into the multivariable logistic regression model, without further stepwise selection. The results showed that these five variables were independent risk factors for postoperative bladder paralysis in CC patients (P < 0.05) (**Table 2**).

**Figure 2:**
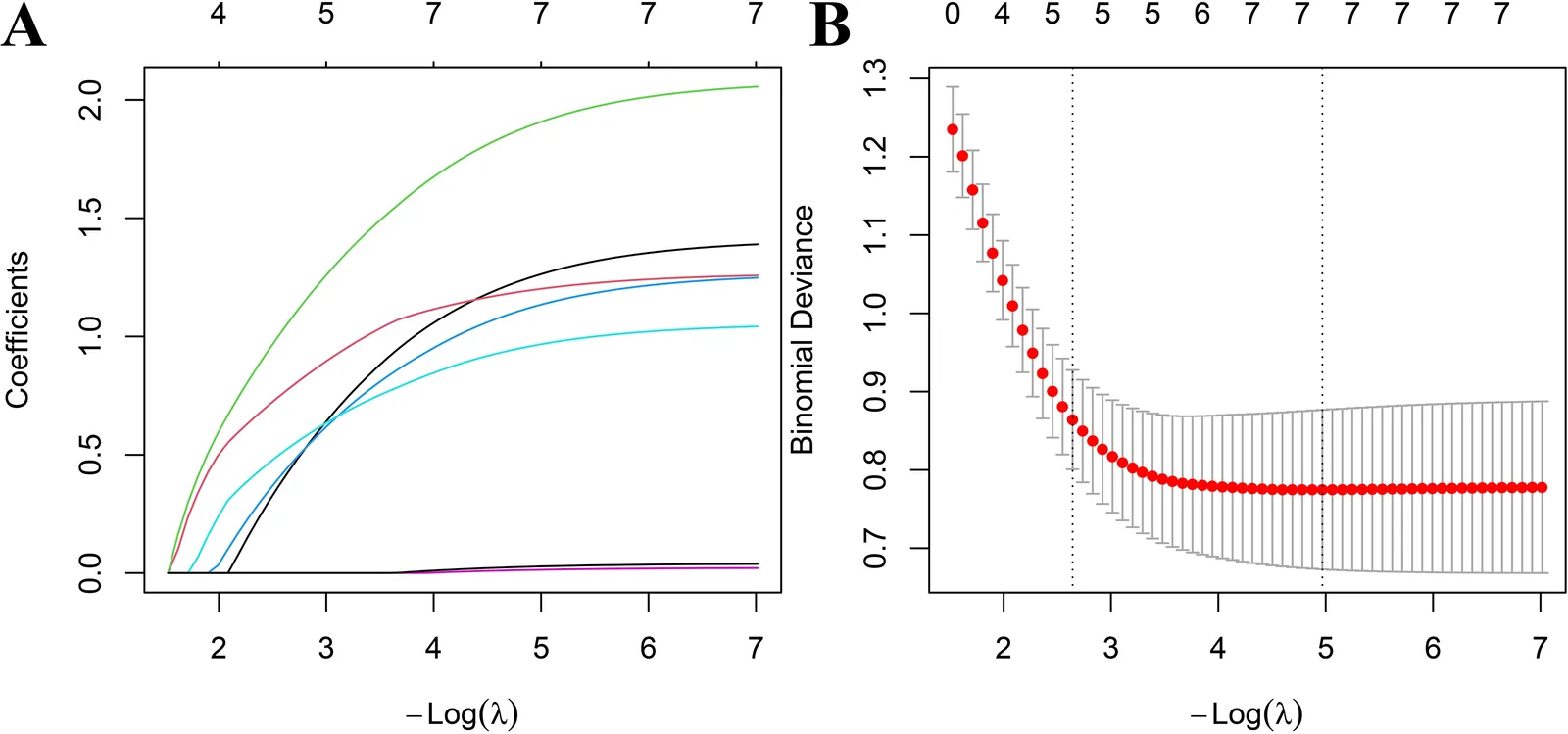
Lasso-logistic regression analysis of risk factors for postoperative bladder paralysis in CC patients. **(A)** Relationship diagram of Lasso regression coefficient; **(B)** Lasso regression 10-fold cross-validation results.

**Table 2:** Lasso-logistic regression analysis of the factors influencing the occurrence of bladder paralysis after surgery in CC patients.

Variable	B value	SE value	Wald $\chi^{2}$ value	*P* value	OR value	95%CI
Clinical staging	1.422	0.435	10.681	0.001	4.145	1.767~9.724
Number of lymph nodes removed	1.396	0.422	10.931	0.001	4.037	1.765~9.235
Extent of hysterectomy	1.439	0.356	16.336	<0.001	4.216	2.098~8.471
Width of parametrial tissue resection	1.184	0.430	7.569	0.006	3.267	1.406~7.594
Vaginal stump length	0.988	0.268	13.602	<0.001	2.687	1.589~4.544
Constant	-3.838	0.480	63.851	<0.001	0.022	–

### 3.3 Construction of a nomogram model for predicting postoperative bladder paralysis in CC patients

Based on the factors screened through Lasso-logistic regression, a nomogram model was constructed with the following formula: P = e^x^/(1 + e^x^), x = -3.838 + 1.422 × (clinical stage) + 1.396 × (number of lymph nodes removed) + 1.439 × (extent of hysterectomy) + 1.184 × (parametrial resection width) + 0.988 × (vaginal stump length). In this model, the most important influencing factor was extent of hysterectomy. A vertical line drawn from each indicator corresponds to a point value, and the total score is the sum of all items. The higher the score, the greater the probability of bladder paralysis. For example, for a patient with stage IIA–IIB, ≥25 lymph nodes removed, modified radical hysterectomy, a parametrial resection width ≥3 cm, and a vaginal stump length ≥3 cm, the linear predictor calculated using the above equation is 1.152, corresponding to a predicted probability of approximately 76.0%. The corresponding point values and total score should be read from the nomogram generated using the final logistic regression coefficients (**Figure 3**).

**Figure 3:**
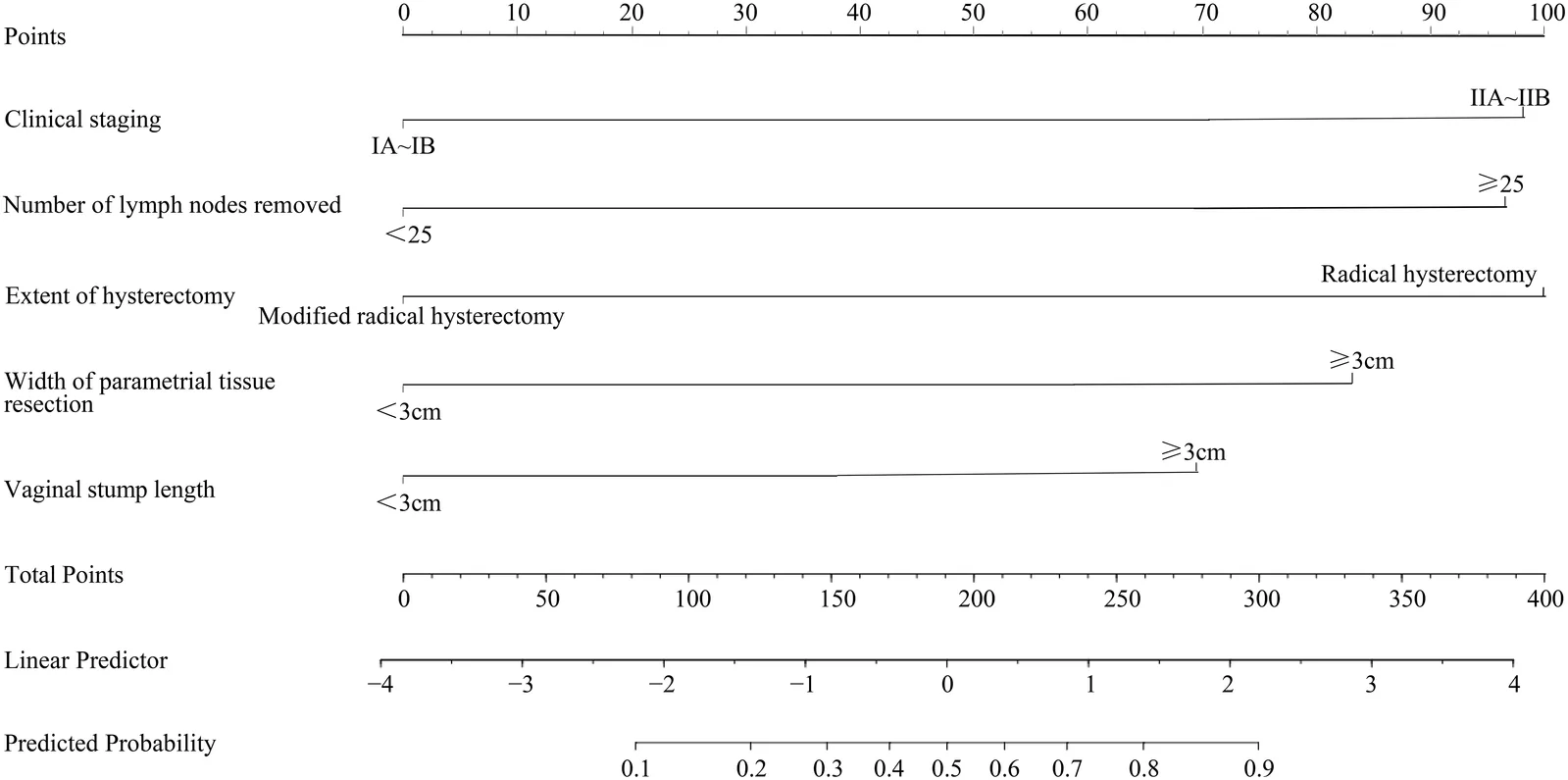
Construct a nomogram model for the occurrence of postoperative bladder paralysis in CC patients.

### 3.4 Internal validation of the prediction model for postoperative bladder paralysis in CC patients

Internal validation showed that the area under the ROC curve (AUC) was 0.899 (95% CI = 0.854–0.946). Internal validation using the Bootstrap method with 1,000 repetitions showed a C-index of 0.899, a calibration intercept of −0.062, a calibration slope of 0.8927, and a Brier score of 0.115.The Hosmer–Lemeshow (H-L) test yielded χ² = 7.405 (P = 0.734), indicating close agreement between predicted and observed probabilities (**Figure 4**).

**Figure 4:**
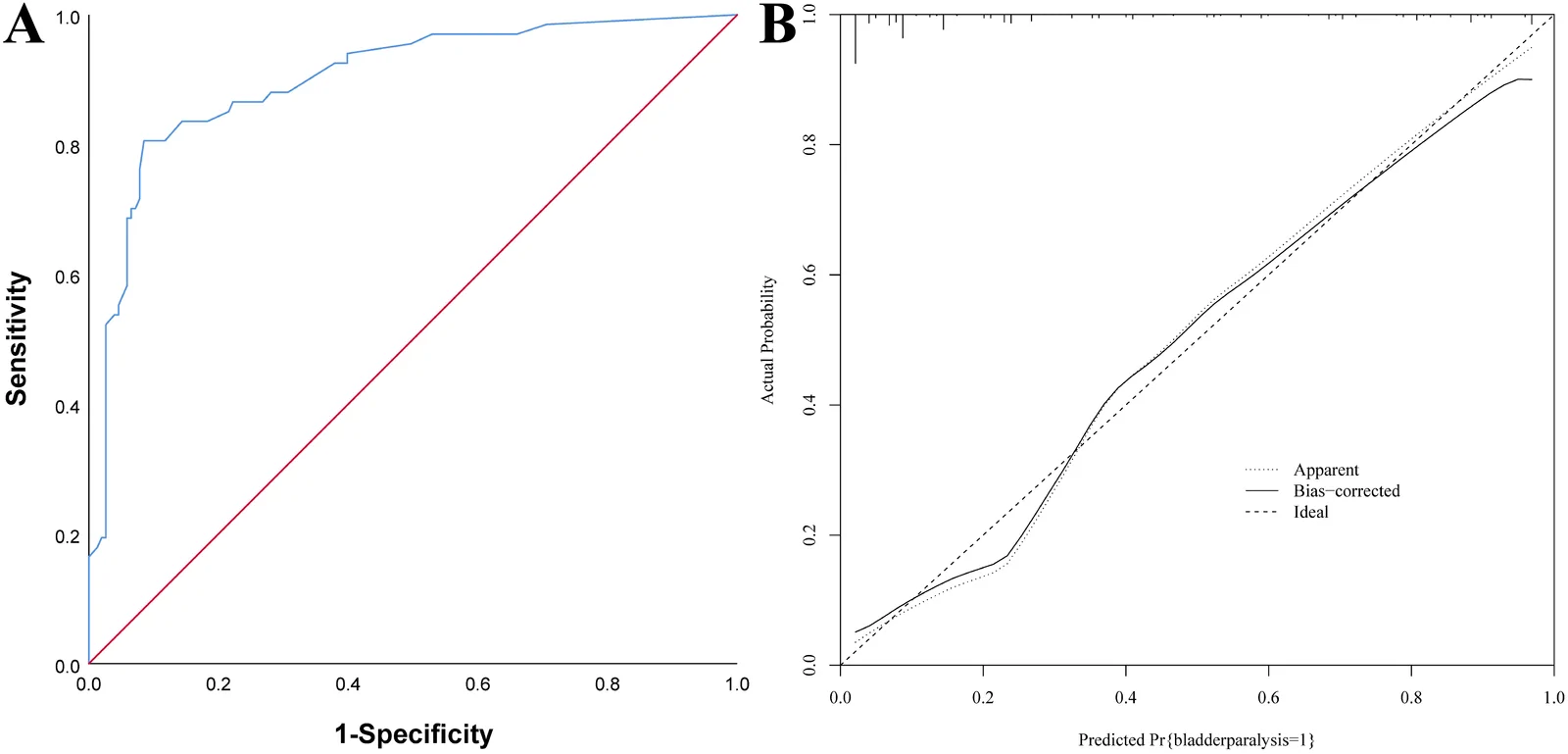
Internal validation of the predictive model of postoperative bladder paralysis among CC patients. **(A)** ROC curves; **(B)** Calibration curves.

### 3.5 Internal validation by DCA curve

Decision curve analysis (DCA) showed that when the threshold probability ranged from 0.14 to 0.82, the net clinical benefit of predicting postoperative bladder paralysis in CC patients was relatively high (**Figure 5**).

**Figure 5:**
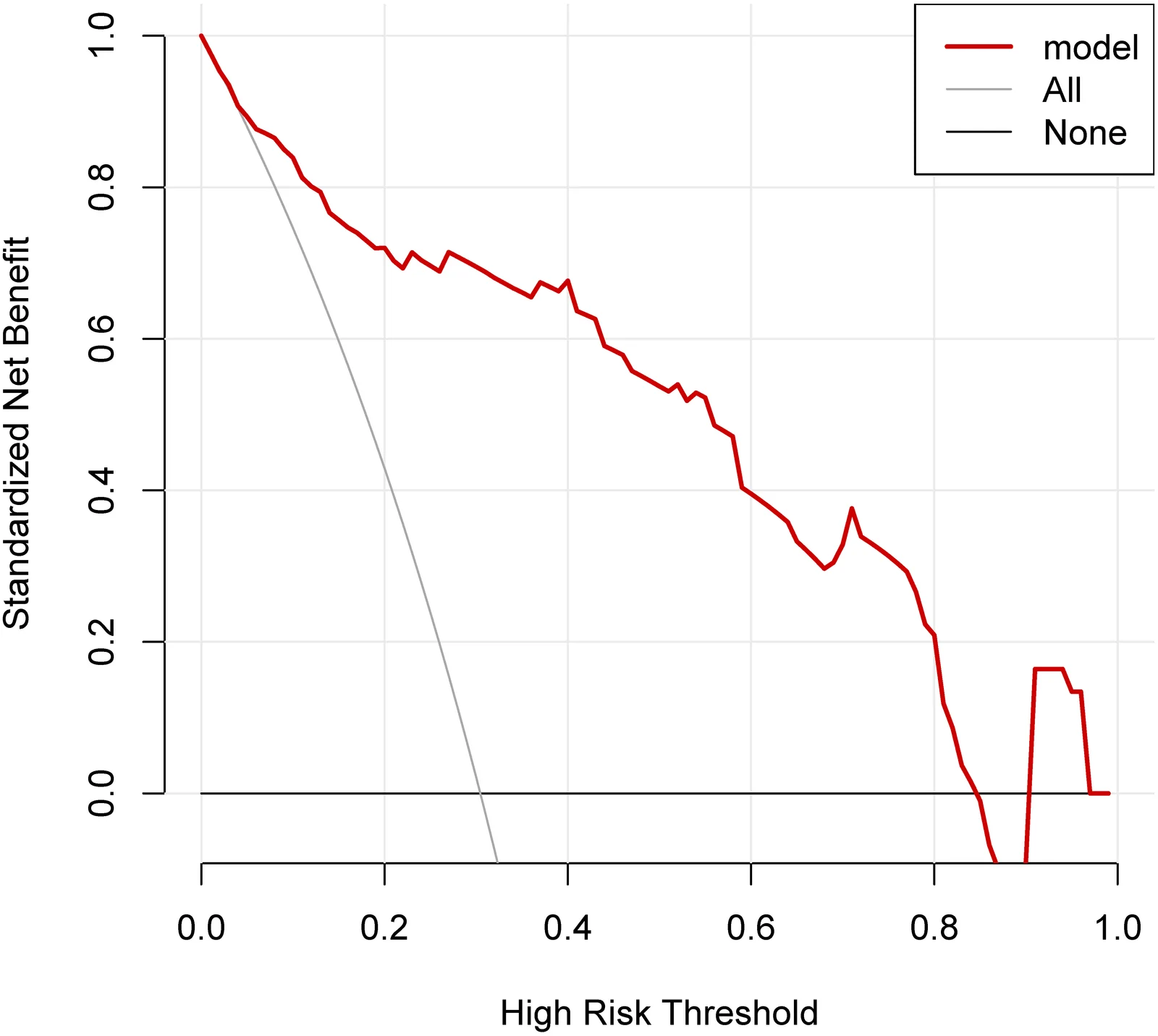
Internal validation of DCA curves.

### 3.6 Comparison of clinical data between the bladder paralysis group and the non-bladder paralysis group in the temporal validation cohort

Among the 130 patients in the temporal validation cohort, 42 developed bladder paralysis (32.31%). Significant differences between the bladder paralysis group and the non-bladder paralysis group were observed in clinical stage, number of lymph nodes removed, extent of hysterectomy, width of parametrial resection, and length of the vaginal stump (P < 0.05). No significant differences were found in other clinical data (P > 0.05) (**Table 3**).

**Table 3:** Comparison of clinical data between the bladder paralysis group and the non-bladder paralysis group in the temporal validation group.

Factor	Bladder paralysis group (n=42)	Non bladder paralysis group (n=88)	*χ* ^2^	*P*
Age			0.000	0.995
≥50 years old	31 (73.81)	65 (73.86)		
<50 years old	11 (26.19)	23 (26.14)		
BMI			0.533	0.465
≥24 kg/m^2^	21 (50.00)	38 (43.18)		
<24 kg/m^2^	21 (50.00)	50 (56.82)		
Diabetes			1.922	0.166
yes	3 (7.14)	14 (15.91)		
no	39 (92.86)	74 (84.09)		
Hypertension			0.986	0.321
yes	14 (33.33)	22 (25.00)		
no	28 (66.67)	66 (75.00)		
Pathological type			0.160	0.689
Squamous cell carcinoma	36 (85.71)	73 (82.95)		
Adenocarcinoma	6 (14.29)	15 (17.05)		
Clinical staging			12.123	<0.001
IA~IB	19 (45.24)	67 (76.14)		
IIA~IIB	23 (54.76)	21 (23.86)		
Postoperative chemoradiotherapy			0.502	0.479
yes	28 (66.67)	53 (60.23)		
no	14 (33.33)	35 (39.77)		
Number of lymph nodes removed (pcs)			7.914	0.005
≥25	36 (85.71)	54 (61.36)		
<25	6 (14.29)	34 (38.64)		
Depth of cervical stromal invasion			2.541	0.111
≥1/2 of the cervical stroma	33 (78.75)	57 (64.77)		
<1/2 of the cervical stroma	9 (21.43)	31 (35.23)		
Amount of bleeding during surgery (mL)			0.657	0.418
≥50	27 (64.29)	50 (56.82)		
<50	15 (35.71)	38 (43.18)		
Surgery time (min)			0.022	0.883
≥180	32 (76.19)	66 (75.00)		
<180	10 (23.81)	22 (25.00)		
Extent of hysterectomy			9.880	0.002
Radical hysterectomy	34 (80.95)	46 (52.27)		
Modified radical hysterectomy	8 (19.05)	42 (47.73)		
Width of parametrial tissue resection (cm)			19.605	<0.001
≥3	25 (59.52)	18 (20.45)		
<3	17 (40.48)	70 (79.55)		
Vaginal stump length (cm)			8.880	0.003
≥3	21 (50.00)	21 (23.86)		
<3	21 (50.00)	67 (76.14)		
Postoperative hospital stay (d)			0.635	0.426
≥7	7 (16.67)	20 (22.73)		
<7	35 (83.33)	68 (77.27)		

### 3.7 Temporal validation of the prediction model for postoperative bladder paralysis in CC patients

Temporal validation using the later cohort from the same institution showed that the AUC of the ROC curve was 0.944 (95% CI = 0.905–0.984). Temporal validation using the Bootstrap method with 1,000 repetitions showed a C-index of 0.944, a calibration intercept of -0.082, a calibration slope of 0.915, and a Brier score of 0.089.The H-L test yielded χ² = 7.234 (P = 0.725), confirming that predicted probabilities were consistent with actual outcomes (**Figure 6**).

**Figure 6:**
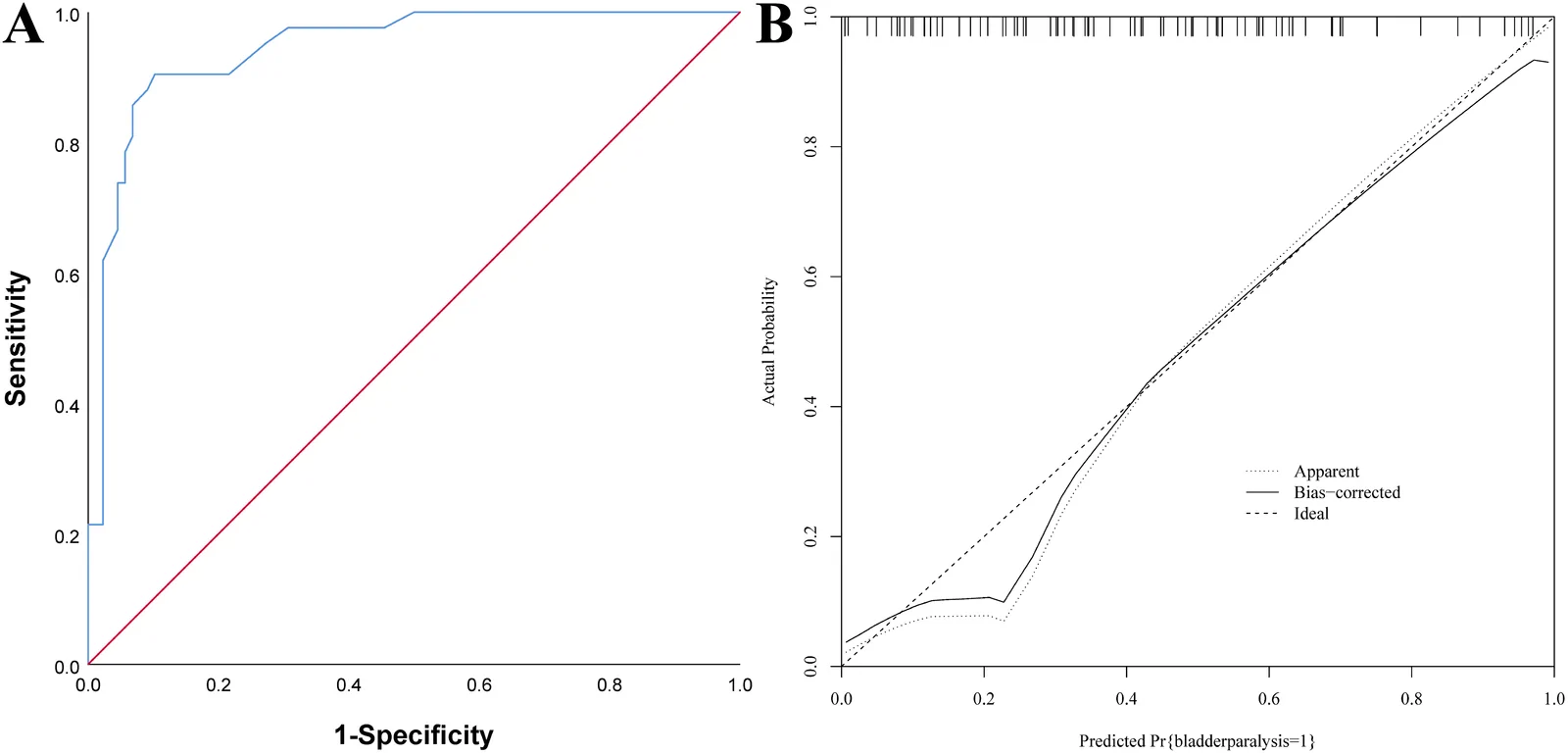
Temporal validation of the predictive model of postoperative bladder paralysis among CC patients. **(A)** ROC curves; **(B)** Calibration curves.

### 3.8 Decision curve analysis in the temporal validation cohort

In the temporal validation cohort,the DCA curve demonstrated that when the threshold probability ranged from 0.19 to 0.84, the net clinical benefit of predicting postoperative bladder paralysis in CC patients was high (**Figure 7**).

**Figure 7:**
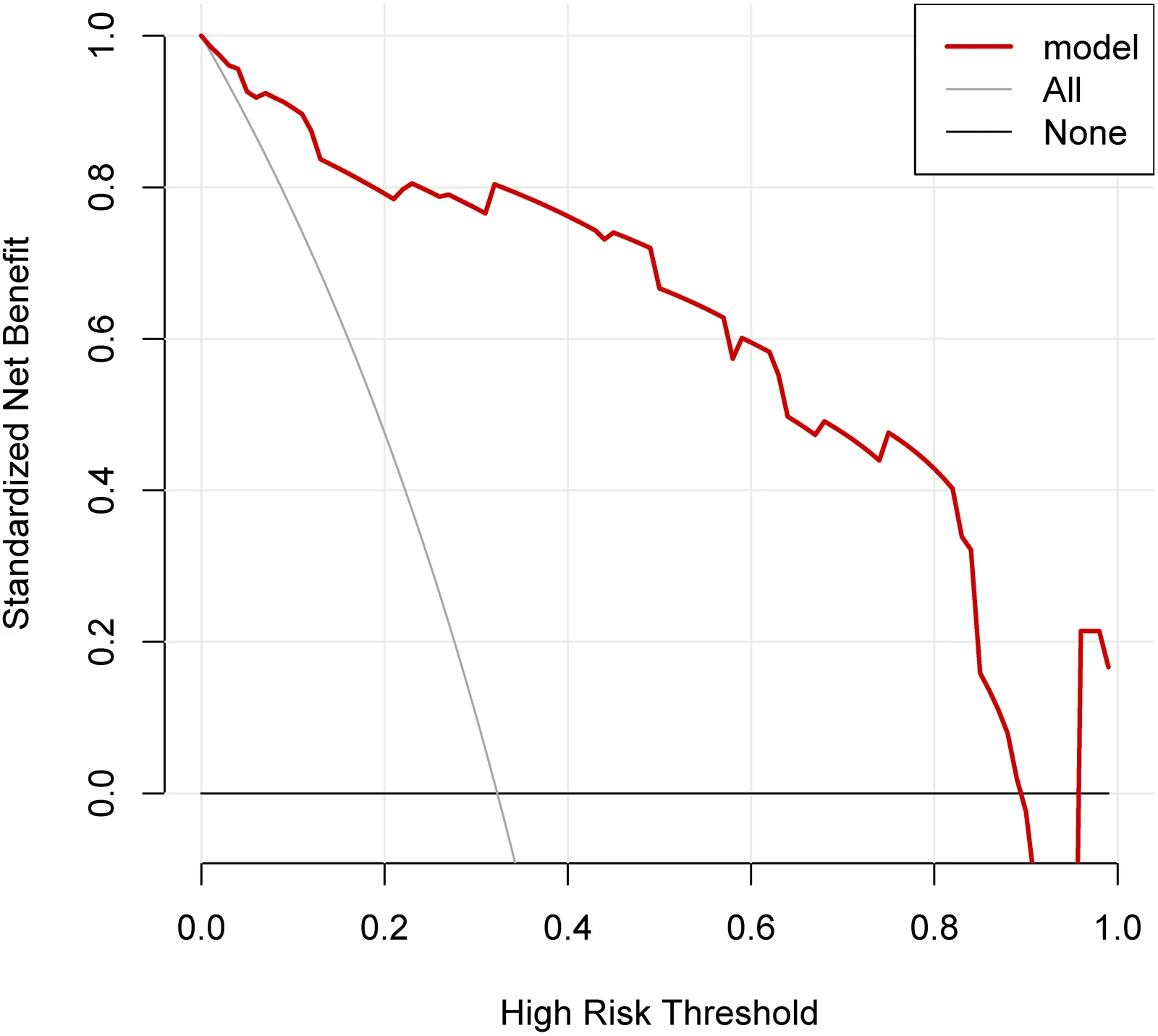
Decision curve analysis in the temporal validation cohort.

## 4 Discussion

At present, surgical treatment remains the mainstay for patients with early-stage CC. However, postoperative complications are common, with bladder paralysis being one of them. During surgery, nerve injury, glandular edema, and damage to the bladder muscular layer may lead to bladder paralysis, which not only affects patients’ quality of life and reduces their confidence in treatment but may also cause upper urinary tract damage and renal failure (11, 12). The results of this study indicate that bladder paralysis is a common complication after surgery. One possible explanation is that surgical procedures often require transection and resection of uterine ligaments and adjacent supporting tissues, leading to positional changes of the bladder and rectum, thereby impairing the structure, function, and integrity of the pelvic floor, which ultimately results in bladder paralysis (13).

Lasso-logistic regression analysis in this study revealed that clinical stage, number of lymph nodes removed, extent of hysterectomy, width of parametrial resection, and length of the vaginal stump were independent risk factors for postoperative bladder paralysis in CC patients. The underlying mechanisms may be explained as follows: (1) Clinical stage: With more advanced clinical stages, the extent of surgery increases, which raises the risk of pelvic nerve injury and thereby the incidence of bladder paralysis (14, 15). (2) Number of lymph nodes removed: The pelvic plexus of the hypogastric nerve innervates the bladder. Lymphadenectomy can damage both superior and inferior bladder innervation. A greater number of lymph nodes removed may result in more nerve damage, aggravating bladder dysfunction and increasing the risk of paralysis (16, 17). (3) Extent of hysterectomy: Radical hysterectomy involves extensive trauma, which may severely damage parasympathetic nerves, surrounding ligaments, and autonomic nerve conduction, thus impairing bladder contraction and sphincter relaxation, making bladder paralysis more likely (18, 19). (4) Width of parametrial resection: During surgery, transection of the cardinal ligament at the pelvic wall and the uterosacral ligament at the sacrum inevitably involves resection of part of the pelvic visceral nerves. This increases the width of parametrial resection and leads to loss of normal sympathetic and parasympathetic innervation of the detrusor muscle. Consequently, peripheral nerve damage occurs, neurotrophic factors for nerve recovery are reduced, and muscle fiber atrophy and dysfunction follow, heightening the risk of bladder paralysis (20, 21). (5) Vaginal stump length: Resection of approximately 3 cm of the upper vaginal segment, coupled with dissection of the ureter and extended parametrial resection, may damage bladder-supporting tissues and blood supply. This can lead to motor and sensory dysfunction, urinary retention, and ultimately increased risk of bladder paralysis (22).

In this study, a nomogram model was constructed based on Lasso regression. The results showed good discrimination, with AUC values of 0.899 and 0.944 in the modeling and temporal validation cohorts, respectively. The Hosmer–Lemeshow test indicated close agreement between predicted and observed probabilities. Decision curve analysis further revealed that when the threshold probabilities were 0.14–0.82 and 0.19–0.84, respectively, the model provided a relatively high net clinical benefit for predicting postoperative bladder paralysis. The favorable performance observed in the later temporal cohort suggests that the model maintained relatively stable predictive performance over time within the same institution. However, because the temporal validation cohort was derived from the same institution as the modeling cohort, its generalizability to other institutions and patient populations remains to be further validated. Recent studies have also demonstrated the value of computational modeling, machine learning-based stratification, and prognostic scoring approaches for cancer patient stratification and individualized risk prediction (23–26),further supporting the application of predictive models in personalized patient management. The model is mainly intended for early postoperative risk stratification of bladder paralysis. For patients identified as high risk by the model, postoperative bladder function monitoring can be intensified, catheter management can be optimized, bladder rehabilitation training can be initiated as early as possible, and focused follow-up of voiding function can be provided, thereby facilitating timely intervention and potentially improving patients’ quality of life.

In conclusion, clinical stage, number of lymph nodes removed, extent of hysterectomy, width of parametrial resection, and length of the vaginal stump are independent risk factors for postoperative bladder paralysis in CC patients. The nomogram model based on Logistic regression demonstrated good calibration and discrimination. However, this study has certain limitations: First, it was a single-center, retrospective study with a relatively small sample size, which may introduce selection bias. Second, the two-stage variable-selection strategy based on univariable screening followed by LASSO regression may introduce additional selection bias and potentially lead to optimistic model performance. Third, although a later cohort from the same institution was used for temporal validation, its generalizability to other institutions and populations remains uncertain. Therefore, independent prospective multicenter external validation with larger sample sizes is warranted.

## Data Availability

The original contributions presented in the study are included in the article/supplementary material. Further inquiries can be directed to the corresponding author.

## References

[1] Jelic MD Mandic AD Maricic SM Srdjenovic BU . Oxidative stress and its role in cancer. J Cancer Res Ther. (2021) 17:22–8. doi: 10.4103/jcrt.jcrt_862_16 33723127

[2] Revathidevi S Murugan AK Nakaoka H Inoue I Munirajan AK . APOBEC: A molecular driver in cervical cancer pathogenesis. Cancer Lett. (2020) 496:104–16. doi: 10.1016/j.canlet.2020.10.004 33038491 PMC7539941

[3] Cao T-T Wen H-W Gao Y-N Lyu Q-B Liu H-X Wang S . Urodynamic assessment of bladder storage function after radical hysterectomy for cervical cancer. Chin Med J (Engl). (2020) 133:2274–80. doi: 10.1097/cm9.0000000000001014 32925291 PMC7546844

[4] Wallin E Falconer H Carlson J Haglund C Koskela LR Radestad AF . Objective and subjective assessment of bladder function after robot-assisted laparoscopic radical hysterectomy for early-stage cervical cancer. J Minim Invasive Gynecol. (2022) 29:1075–82. doi: 10.1016/j.jmig.2022.05.018 35654357

[5] Bereza K Kukiełka A Trojnarska D Grabarek BO Opławski M Banaś T . Multidimensional assessment of urinary dysfunction and quality of life after cervical cancer treatment: a multicenter study integrating urodynamics and patient-reported outcomes. Front Oncol. (2026) 16:1853725. doi: 10.3389/fonc.2026.1853725 42465566 PMC13372604

[6] Yang S Liu C Li C Hua K . Nomogram predicting lymph node metastasis in the early-stage cervical cancer. Front Med (Lausanne). (2022) 9:866283. doi: 10.3389/fmed.2022.866283 35847788 PMC9280490

[7] Wu J Guo Q Zhu J Wu Y Wang S Liang S . Developing a nomogram for preoperative prediction of cervical cancer lymph node metastasis by multiplex immunofluorescence. BMC Cancer. (2023) 23:485. doi: 10.1186/s12885-023-10932-0 37254049 PMC10228122

[8] Saleh M Virarkar M Javadi S Elsherif SB Faria SDC Bhosale P . Cervical cancer: 2018 revised International Federation of Gynecology and Obstetrics staging system and the role of imaging. AJR Am J Roentgenol. (2020) 214:1182–95. doi: 10.2214/ajr.19.21819 32182097

[9] Reed N Balega J Barwick T Buckley L Burton K Eminowicz G . British Gynaecological Cancer Society (BGCS) cervical cancer guidelines: Recommendations for practice. Eur J Obstet Gynecol Reprod Biol. (2020) 256:433–65. doi: 10.1016/j.ejogrb.2020.08.020 33143928

[10] Laterza RM Sievert K-D De Ridder D Vierhout ME Haab F Cardozo L . Bladder function after radical hysterectomy for cervical cancer. Neurourol Urodyn. (2014) 34:309–15. doi: 10.1002/nau.22570 24519734

[11] Zhao Y-T Guo S Shang Z-C Ding H-Y Jin J-H Chu K-Y . Study on the recovery of bladder function in patients with cervical cancer after operation by portable ultrasound combined. Int J Artif Organs. (2024) 47:659–65. doi: 10.1177/03913988241262593 39076041

[12] Zhao Q . Application and clinical efficacy of comprehensive nursing intervention in pelvic floor and bladder function recovery post-cervical cancer surgery. Pak J Med Sci. (2024) 40:2287–92. doi: 10.12669/pjms.40.10.9271 39554685 PMC11568732

[13] Baessler K Windemut S Chiantera V Köhler C Sehouli J . Sexual, bladder and bowel function following different minimally invasive techniques of radical hysterectomy in patients with early-stage cervical cancer. Clin Transl Oncol. (2021) 23:2335–43. doi: 10.1007/s12094-021-02632-7 34003456 PMC8455389

[14] Carter J Huang HQ Monk BJ Kim Y-B Kim M-H Stuckey A . Evaluation of physical function and quality of life before and after nonradical surgical therapy for stage IA1 and IA2-IB1 cervical cancer (GOG-0278). Gynecol Oncol. (2025) 195:50–8. doi: 10.1016/j.ygyno.2025.02.023 40058184 PMC12009199

[15] Zhang J Zhang Y Liu J Liu J Liu M Chang R . The impact of early postoperative pelvic floor muscle exercise on urinary retention and urodynamics after radical hysterectomy for cervical cancer. Arch Esp Urol. (2025) 78:579–87. doi: 10.56434/j.arch.esp.urol.20257805.78 40641168

[16] Ślusarczyk A Scilipoti P Marcq G Pradere B Contieri R Krajewski W . Comparison of extended and standard lymph node dissection in radical cystectomy for urothelial bladder cancer: a systematic review and meta-analysis. World J Urol. (2025) 43:181. doi: 10.1007/s00345-025-05549-w 40111501

[17] Jiang S Jiang P Jiang T Tu Y Zhang J Li N . Effect of number of retrieved lymph nodes on prognosis in FIGO stage IA1-IIA2 cervical cancer patients treated with primary radical surgery. Clin Med Insights Oncol. (2022) 16:11795549221127161. doi: 10.1177/11795549221127161 36199803 PMC9528045

[18] Terada S Terai Y Tanaka Y Tanaka T Tsunetoh S Ohmichi M . Postsurgical urodynamic study of total laparoscopic nerve-sparing radical hysterectomy for uterine cervical cancer. J Obstet Gynaecol Res. (2022) 48:2863–71. doi: 10.1111/jog.15371 35934761

[19] Zong J You M Li C . Effect of Kegel pelvic floor muscle exercise combined with clean intermittent self-catheterization on urinary retention after radical hysterectomy for cervical cancer. Pak J Med Sci. (2022) 38:462–8. doi: 10.12669/pjms.38.3.4495 35480547 PMC9002440

[20] Plante M Kwon JS Ferguson S Samouëlian V Ferron G Maulard A . Simple versus radical hysterectomy in women with low-risk cervical cancer. N Engl J Med. (2024) 390:819–29. doi: 10.1056/nejmoa2308900 38416430

[21] Ramirez PT Robledo KP Frumovitz M Pareja R Ribeiro R Lopez A . LACC trial: Final analysis on overall survival comparing open versus minimally invasive radical hysterectomy for early-stage cervical cancer. J Clin Oncol. (2024) 42:2741–6. doi: 10.1200/jco.23.02335 38810208

[22] Wang Z Zeng A Long F Wu M Tan X-J Liu Z-W . Use of vaginal reconstructive surgery in cervical cancer patients to prevent vaginal stump contracture. J Invest Surg. (2019) 34:747–53. doi: 10.1080/08941939.2019.1683658 31739704

[23] Lu Q Wang S Pan Y Yu Y Xiong Y Wei H . The immune-based prognostic score for the immunogenomic landscape aanalysis and application of chemotherapy in breast cancer. Curr Bioinf. (2022) 17:624–31. doi: 10.2174/1574893617666220524123825

[24] Su D Li H Wang T Zou M Wei H Xiong Y . Integrated somatic mutation network diffusion model for stratification of breast cancer into different metabolic mutation subtypes. Curr Bioinf. (2025) 20:246–56. doi: 10.2174/0115748936298012240322091111

[25] Xiong Y Li S Huo Z Zou M Su D Li H . Integrated metabolic-related transcription factor protein activity for stratification of breast cancer with distinct clinical outcomes. Curr Bioinf. (2026) 21:407–21. doi: 10.2174/0115748936380191250710051557

[26] He Y Wei H Liao S Ou R Xiong Y Zuo Y . Integrated machine learning algorithms for stratification of patients with bladder cancer. Curr Bioinf. (2024) 19:963–76. doi: 10.2174/0115748936288453240124082031

